# Do Differences in Chemical Composition of Stem and Cap of *Amanita muscaria* Fruiting Bodies Correlate with Topsoil Type?

**DOI:** 10.1371/journal.pone.0104084

**Published:** 2014-12-01

**Authors:** Stanisław Deja, Piotr P. Wieczorek, Marek Halama, Izabela Jasicka-Misiak, Paweł Kafarski, Anna Poliwoda, Piotr Młynarz

**Affiliations:** 1 Faculty of Chemistry, Opole University, Opole, Poland; 2 Museum of Natural History, Wrocław University, Wrocław, Poland; 3 Department of Bioorganic Chemistry, Wrocław University of Technology, Wrocław, Poland; Institute for Sustainable Plant Protection, C.N.R., Italy

## Abstract

Fly agaric (*Amanita muscaria*) was investigated using a ^1^H NMR-based metabolomics approach. The caps and stems were studied separately, revealing different metabolic compositions. Additionally, multivariate data analyses of the fungal basidiomata and the type of soil were performed. Compared to the stems, *A. muscaria* caps exhibited higher concentrations of isoleucine, leucine, valine, alanine, aspartate, asparagine, threonine, lipids (mainly free fatty acids), choline, glycerophosphocholine (GPC), acetate, adenosine, uridine, 4-aminobutyrate, 6-hydroxynicotinate, quinolinate, UDP-carbohydrate and glycerol. Conversely, they exhibited lower concentrations of formate, fumarate, trehalose, α- and β-glucose. Six metabolites, malate, succinate, gluconate, *N*-acetylated compounds (NAC), tyrosine and phenylalanine, were detected in whole *A. muscaria* fruiting bodies but did not show significant differences in their levels between caps and stems (*P* value>0.05 and/or OPLS-DA loading correlation coefficient <0.4). This methodology allowed for the differentiation between the fruiting bodies of *A. muscaria* from mineral and mineral-organic topsoil. Moreover, the metabolomic approach and multivariate tools enabled to ascribe the basidiomata of fly agaric to the type of topsoil. Obtained results revealed that stems metabolome is more dependent on the topsoil type than caps. The correlation between metabolites and topsoil contents together with its properties exhibited mutual dependences.

## Introduction

Although the functional importance of fungi in ecosystems [1], [2] with the food and beverage industries, agriculture, the pharmaceutical and agrochemical industries, and medicine being regularly emphasized [3], [4], there has been comparatively little focus on fungal metabolic properties in various wild habitats. Soil conditions have a profound influence, directly or indirectly, on the distribution of terrestrial macrofungi [5]–[10]. Mushrooms are an important source of biologically active compounds of medicinal value. However, relatively little is known about the distribution of low molecular weight metabolites within these organisms (in caps and stems) and its relationship with the edaphic physical and chemical properties of the topsoil. Thus, it is important to better understand the metabolic profile of mushrooms and the macromycetes response to topsoil characteristics.

*Amanita muscaria*, commonly known as fly agaric or fly amanita, is one of the most gorgeous members of the basidiomycetous genus *Amanita* and also one of the most striking and recognizable of all macrofungi [11], [12]. This inedible, neurotropic mushroom [13]–[15] is native to temperate and boreal regions of the Northern Hemisphere; however, it has also been unintentionally introduced to many countries in the Southern Hemisphere, and it seems to have become a cosmopolitan species [16]–[28]. *A. muscaria* forms symbiotic ectomycorrhizal associations with a broad range of hosts, including those from the families *Betulaceae, Cistaceae, Cupressaceae, Fagaceae, Pinaceae, Rosaceae* and *Salicaceae*, and it associates most frequently with tree members of genera *Betula*, *Pinus* and *Picea* [29]–[33]. It appears that the typical form of fly agaric (*A. muscaria* var. *muscaria*), which can be found in a variety of habitats in Europe, North Asia, and the most northwestern parts of North America (Alaska), is straightforward to identify. Nevertheless, *A. muscaria* can occur in different forms and colors. For that reason, varieties (some considered subspecies or geographical races) with differing cap color from various regions of the Northern Hemisphere have been described [34], [35]. Furthermore, in recent years, molecular tools have revealed phylogenetic speciation within fly amanita, which had previously been treated as one morphological species. Genetic studies conducted by Oda et al. [36] and Geml et al. [37]–[39] showed that *A. muscaria* is composed of several unique major lineages that appear to be distinct phylogenetic species, with no gene flow occurring among them.

The contents and distribution of toxins (α-amanitin, β-amanitin, γ-amanitin, amaninamide, desoxoviroidin, phallisin, phallacidin, phallisacin, phalloidin, phalloin) in various tissues (cap, gills, ring, stipe, bulb, volva), spores and developmental stages of several ectomycorrhizal species such as *Amanita bisporigera, Amanita exitialis, Amanita fuliginea, Amanita subjunquillea* and *Amanita phalloides* have been extensively investigated [40]–[45]. Substantial differences in the tissue toxin content were revealed [40], [42]–[44] and the differences in contents and distribution of some individual toxins in the tissues related to the carpophore developmental stage was also underlined [40], [43], [46]. Furthermore, the possible influence of the collection site and the collection date on the toxin composition, their time stability and the tissue distribution, as the example of *A. phalloides* carpophores have been also analysed [44], [45]. These investigations indicated that environmental factors and mainly the soil type can clearly affect the amatoxin and phallotoxin composition of *A. phalloides* carpophores, including their histologically different parts.

According to our knowledge, no substantial attempts have been made to relate metabolic fingerprints of *A. muscaria* to the main structural parts of the mature fruiting body or to determine their correlation with the physico-chemical properties of the microhabitat. Therefore, a metabolomic approach was used to study fly agaric collected in southwestern Poland, with the purposes of improving the knowledge of its chemical composition and determining whether the compounds correlate quantitatively or qualitatively with the topsoil properties or the structural parts of the mature fruiting bodies. The following hypotheses were examined: a) the levels of metabolites in *A. muscaria* vary with the structure (cap or stem) of the mature fruiting body, b) the soil at the collection site, due to microhabitat factors (chemical element content, acidity, humus and total carbon content), influences the metabolic fingerprint of the *A. muscaria* fruiting body.

## Materials and Methods

### Sample sites, mushrooms and soil sampling

**Figure 1:**
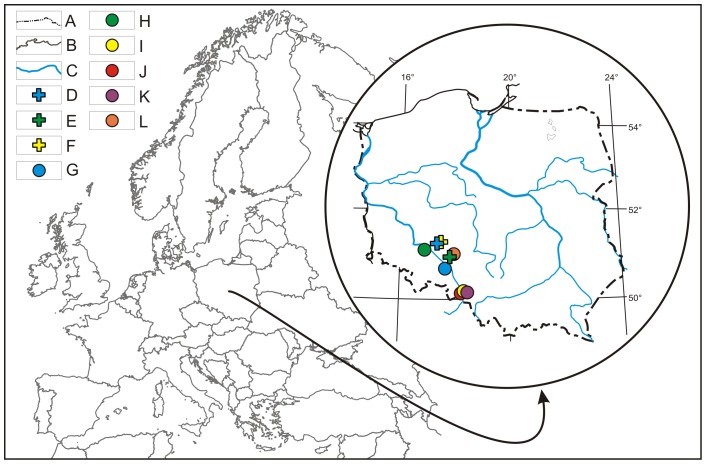
Map showing the distribution of sampling sites. A: border of countries, B: border of Poland, C: main rivers, D–F: sites on organic-mineral soils (blue: 82, 83; yellow: 72, 73, 75; green: 105), G–L: sites on mineral soils (red: 136, 138, 141; yellow: 129, 131; purple: 275, 278, 280; blue: 120, 121; orange: 96, 98, 102; green: 161, 165, 169). Colours indicate the collection number.

*A. muscaria* fruiting bodies were collected from June–October (20010–2011) from nine sites representing various macrohabitats in southwestern Poland (**Table 1**, **Figure 1**). Within these sites, representative areas of 30×30 m constituted the sampling plots for soil and basidiomata. The collected basidiomata varied in age and size, but very old or damaged specimens were rejected. Fresh basidiomata were cleaned using a plastic knife to remove adherent plant and substrate debris, and the bottom parts of the stems were cut away. Then, they were air-dried with an electric desiccator at 40°C for 24 h and stored in plastic bags for chemical analysis. Additionally, 27 soil samples (9 sites x 3 replicates each) of the upper forest soil horizon (0–20 cm) were collected after removing the surface layer. The soil substrate samples were stored in plastic bags and air-dried at room temperature in clean conditions for a few weeks for further chemical analysis.

**Table 1:** Site characteristics.

Collection number	Site name	Collection date	Macrohabitat type	Associated tree species	Soil type
96, 98, 102	Domaszowice51.04110000°N17.89668300°E	19 Sep. 2010	lawn planted with trees/shrubs (petrol station area)	*Betula pendula*, *Picea abies*, *Larix decidua*	mineral
105	Domaszowice51.03173717°N17.88173780°E	19 Sep. 2010	woodland and scrub	*Betula pendula*, *Picea abies*, *Pinus sylvestris*	org.-mineral
129, 131	Babice50.12896600°N50.12896600°N	24 Sep. 2010	woodland	*Betula pendula*	mineral
120, 121	Borkowice/Skorogoszcz50.74630667°N17.70678833°E	24 Sep. 2010	woodland	*Betula pendula*	mineral
275, 278, 280	Nędza50.15218000°N18.32316167°E	08 Oct. 2011	woodland and scrub	*Betula pendula*, *Pinus sylvestris*	mineral
136, 138, 141	Raszczyce50.12373000°N18.29080500°E	24 Sep. 2010	fallow land/field	*Betula pendula*, *Corylus avellana*, *Pinus sylvestris*, *Quercus robur*	mineral
82, 83	Sokołowice51.26962216°N17.48873212°E	15 Sep. 2010	woodland and scrub	*Betula pendula*, *Pinus sylvestris*	org.-mineral
72, 73, 75	Twardogóra51.32662931°N17.51664770°E	15 Sep. 2010	woodland and scrub	*Betula pendula*, *Pinus sylvestris*	org.-mineral
161, 165, 169	Wrocław51.15440000°N16.94238300°E	23 Sep. 2010	fallow land/field planted with trees/shrubs	*Betula pendula*, *Quercus sp*.	mineral

### Topsoil analysis

All metals were quantified using ASA 1100B (Perkin-Elmer), except for Mg, which was analyzed using ASA Avanta ∑ (GBC), and Hg, which was analyzed using AMA 254 (ALTEC). The measurements were performed in a commercial certified laboratory according to Polish standards: PB 1 ed. 2, PB 2 ed.4, PB 4 ed. 3, PB 5 ed.2, PB 65 ed. 1, PN-R-04016:1992, PN-R-04017:1992, PN-R-04018:1993, PN-R-04019:1993, PN-R-04020:1994 +Az1:2004, PN-R-04021:1994, PN-R-04022:1996+Az1:2002, PN-R-04023:1996.

### Sample preparation for NMR spectroscopy

A total of 22 fungi samples were used in this study. The analyzed carpophores represented mature, fully-developed specimens – comparable by their development stage and sizes. Each fruiting body was divided into cap and stem sections, resulting in 44 paired samples. Weighed tissue (**Table S1**) was transferred into a steal bead homogenizer (Tissuelyser LT; QIAGEN, Hilden, Germany) and disrupted for 10 minutes with shaking at 50 Hz. The resulting powder was suspended in 1 mL of 0.25 M phosphate buffer solution (PBS), pH = 7.4, containing 10% D_2_O, 3 mM sodium azide and 1 mM sodium salt of trimethylsilyl-2,2′,3,3′-tetradeuteropropionic acid (TSP), which was used as an internal standard. The solutions were again shaken for 10 minutes at the same frequency and placed in an ultrasound bath at ambient temperature for 10 minutes. To remove cellular debris and substances that are insoluble in PBS, the samples were centrifuged at 15000×g for 20 minutes. Supernatant aliquots of 500 µL were transferred into 5 mm NMR tubes prior to the measurements.

### ^1^H NMR measurements

All NMR spectra were recorded with an NMR spectrometer (Bruker Biospin Avance II; Bruker, GmBH, Germany) operating at a proton frequency of 600.58 MHz. ^1^H NMR Carr-Purcell-Meiboom-Gill (CPMG) pulse sequence with water suppression was utilized to filter out broad spectral resonances that could have arisen from protein content (number of loops  = 80, spin echo delay  = 400 µs). The spectra were acquired at 300 K after the samples were kept inside the spectrometer for at least 3 minutes. For each sample, 128 scans were collected, resulting in 64 k data points and a spectral width of 20.01 ppm. All spectra were manually phased and baseline-corrected using Topspin 1.3 software (Bruker, GmBH, Germany) with reference to the TSP signal (δ = 0 ppm). Signal assignments were performed based on in-house and online (HMDB, BMRB) databases and confirmed using two-dimensional NMR experiments, ^1^H-^1^H COSY and ^1^H-^1^H TOCSY.

### Multivariate data analysis

Prior multivariate data analysis spectra were preprocessed. Data reduction was performed by binning the spectra into 0.001 ppm-width buckets (region 0.5–10.0 ppm), leading to 9,251 variables after excluding the residual water signal region (4.70–4.95 ppm). The obtained dataset (X matrix) was normalized by dividing each spectral bucket by the corresponding sample mass, and therefore, signal areas should reflect metabolite abundances relative to the fungal weight. Such a transformed data matrix was the input for SIMCA-P+ software (v 13.0, Umetrics, Umeå, Sweden), where principal component analysis (PCA), partial least-squares discriminant analysis (PLS-DA) and orthogonal partial least-squares discriminant analysis (OPLS-DA) were performed. The data were scaled using Pareto scaling.

### Statistical analysis

Univariate statistical analysis was performed using a Mann-Whitney-Wilcoxon (MWW) test. All statistical results were evaluated at α = 0.05. In addition to the *P* value from the MWW test, an absolute value of the OPLS-DA correlation loading (*p(corr)*>0.4) was used as a criterion for significance. Statistical computations employed the Statistica software package (v 10, StatSoft, Tulsa, USA). The Spearman rank correlation coefficient was used to observe relationships between soil factors and metabolite contents in each structure of the basidiomata (stems and caps). The heat maps were generated using MATLAB.

### Ethics statement

No specific permits were required for the field studies apart from that from The Main Pharmaceutical Inspector of the Republic of Poland for the collecting and processing of psychotropic chemicals. This permit was obtained for the described field and laboratory studies (permission number: GIF-N-P/4420/6/09). The field of this studies did not involve endangered or protected species.

## Results

### Discrimination between stems and caps of *A. muscaria* basidiocarp

**Figure 2:**
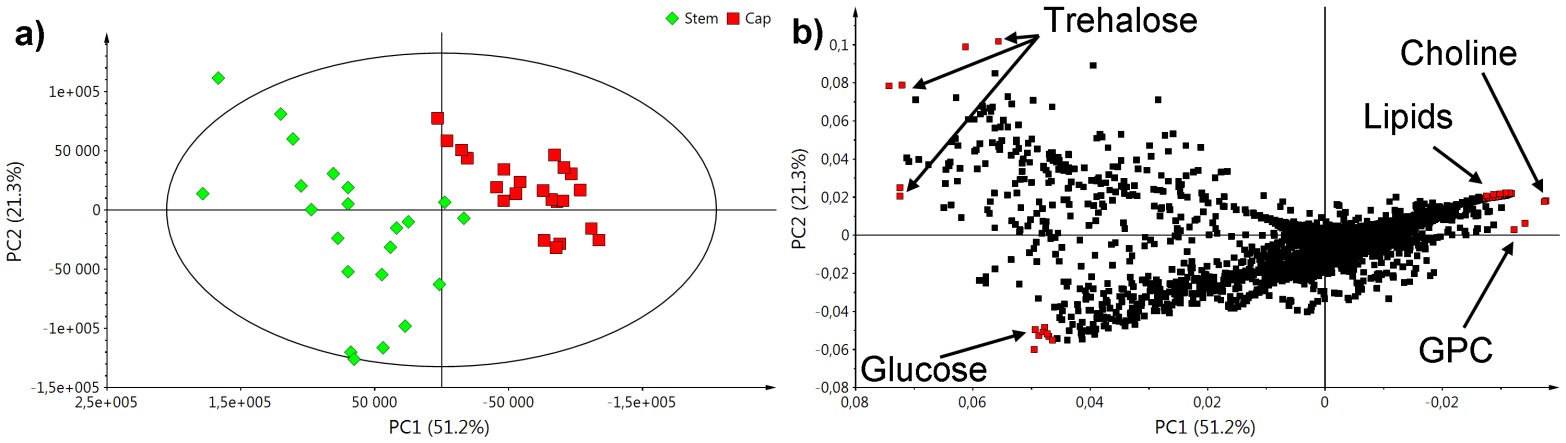
PCA (*R* ^2^ *X* = 0.725; *Q* ^2^ *Y* = 0.671) results: a) score plot; b) corresponding loadings plot.

Data reduction and visualization were performed using PCA (two principal components; *R* ^2^ *X* = 0.725; *Q* ^2^ *Y* = 0.671). A score plot of the first two principal components (PCs) showed a clear grouping of the *A. muscaria* fruiting body parts, namely caps and stems (**Figure 2a**). The caps were much more homogeneous group than stems. The first principal component (PC1) corresponded to 51.2% of the total variance and was responsible for the separation observed between stems and caps. In contrast, the second principal component (PC2) corresponded to 21.3% of the total variance and could be assigned to intra-group variability, mainly in stems. Loadings plot (**Figure 2b**) revealed that lipids, choline and glycerophosphocholine (GPC) mainly contributed to PC1, while glucose and trehalose were greatest constituents of PC2.

**Figure 3:**
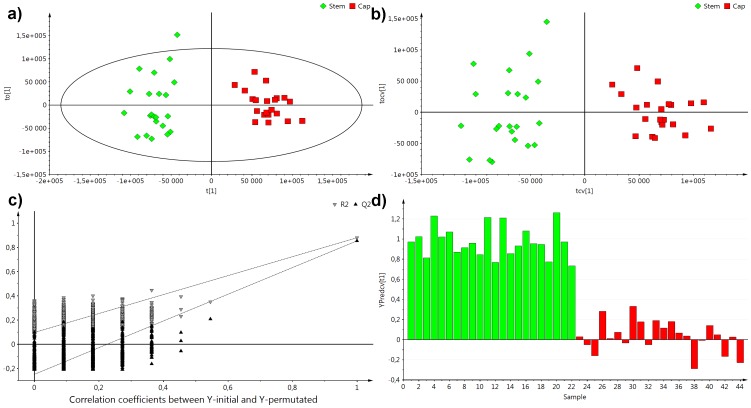
OPLS-DA (*R* ^2^ *X* = 0.774, *R* ^2^ *Y* = 0.938 and *Q* ^2^ *Y* = 0.904) results obtained for discrimination between stems and caps of *A. muscaria* basidiocarp: a) score plot obtained from OPLS-DA model; b) cross-validated score plot obtained from the OPLS-DA model; c) 999 permutations validation test; d) bar plot of cross-validated Y-predicted values from the OPLS-DA model.

**Figure 4:**
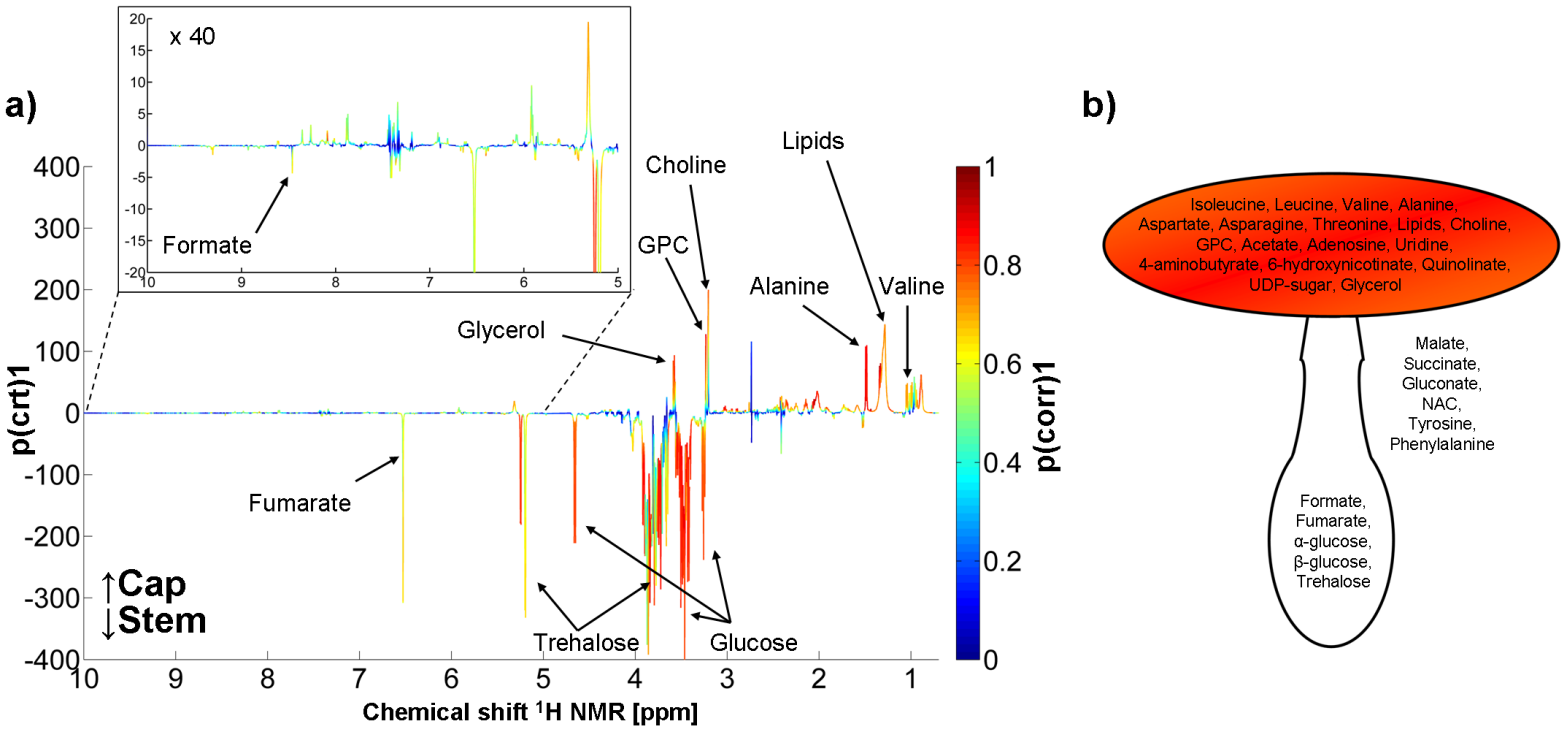
Distribution of metabolites in *A. muscaria* basidocarps: a) loadings of discrimination between stems and caps from the OPLS-DA model. The color bar corresponds to the absolute value of the correlation loading; b) simplified representation of metabolite distributions in *A. muscaria* basidocarps.

To identify the regions of the fingerprint that are the most different between stems and caps, the discriminant variant of OPLS analysis was utilized. Again, as in the case of PCA, the model showed full separation between the analyzed groups and good model parameters (*R* ^2^ *X* = 0.774, *R* ^2^ *Y* = 0.938 and *Q* ^2^ *Y* = 0.904) (**Figure 3a**). The OPLS-DA results were validated by checking cross-validated score plots (internal cross validation CV1) (**Figure 3b**), 999 permutation test (**Figure 3c**) and cross-validated Y predicted values (**Figure 3d**), which again showed full separation of the examined samples and provided evidence that the model is stable. Loading plots were used to evaluate the influence of particular metabolites on the model's discrimination ability. Generally, carbohydrates were present at higher concentrations in stems, while an aliphatic region revealed many metabolites of higher concentrations in caps (**Figure 4a**). Metabolites that were identified and further tested using univariate statistical analysis are listed in **Table 2**. *A. muscaria* caps were characterized by higher concentrations of several amino acids (AAs) including isoleucine, leucine, valine, alanine, aspartate, asparagine, and threonine, as well as lipids, glycerol, choline and GPC compared to stems. Elevated levels of acetate, adenosine, uridine, 4-aminobutyrate, 6-hydroxynicotinate, qinolinate and UDP-carbohydrate were also observed in caps. Conversely, the stems had higher concentrations of formate and fumarate, and carbohydrates, namely α- and β-glucose and trehalose. Six metabolites: malate, succinate, gluconate, *N*-acetylated compounds (NAC), tyrosine and phenylalanine, were detected in whole *A. muscaria* fruiting bodies but did not shown significant differences between caps and stems (*P* value>0.05) and/or OPLS-DA loading correlation coefficients (*p(corr)* <0.4). A simplified representation of metabolite distributions in *A. muscaria* basidocarps is presented in **Figure 4b**.

**Table 2:** Metabolic differences between stems and caps of *A. muscaria* basidocarps.

No	Compound	^1^H NMR: δ, (multiplicity*)	RSD [%]		Cap vs. Stem		
			Stem	Cap	difference[%]	OPLS-DA absolute correlation coefficient	*P* value
1	Isoleucine	0.94 (t); 1.01 (d); 1.27 (m); 1.47 (m); 1.98 (m)	30.6	46.3	116.7	0.716	0.00000
2	Leucine	0.96 (d); 0.97 (d)	53.3	64.9	58.4	0.492	0.03268
3	Valine	1.00 (d); 1.05 (d); 2.26 (m); 3.61 (d)	29.2	63.0	303.6	0.709	0.00000
4	Alanine	1.49 (d); 3.79 (q)	40.1	31.5	294.8	0.869	0.00000
5	Aspartate	2.70 (dd); 2.81 (dd); 3.92 (dd)	24.1	21.3	41.2	0.790	0.00002
6	Asparagine	2.88 (dd); 2.96 (dd); 4.00 (dd)	35.7	21.5	41.6	0.842	0.00041
7	Threonine	1.34 (d); 3.60 (d); 4.26 (m)	29.1	20.7	57.3	0.755	0.00000
8	Tyrosine	6.90 (d); 7.20 (d)	57.0	52.1	54.6	0.288	0.00335
9	Phenylalanine	7.34 (m); 7.39 (m); 7.44 (m)	39.8	53.0	10.4	0.382	0.75374
10	4-aminobutyrate	1.92 (m); 2.30 (t); 3.02 (t)	33.5	25.4	62.8	0.688	0.00001
11	NAC#	2.08 (s)	34.8	24.7	27.1	0.175	0.00335
12	Acetate	1.93 (s)	19.3	26.3	29.7	0.419	0.00089
13	Succinate	2.41 (s)	38.4	27.8	-1.6	0.393	0.89833
14	Malate	2.38 (dd); 2.68 (dd); 4.31 (dd)	35.0	31.2	-20.6	0.333	0.04148
15	Fumarate	6.53 (s)	63.7	43.6	-63.8	0.585	0.00001
16	Formate	8.46 (s)	40.3	50.0	-38.3	0.551	0.00260
17	UDP-carbohydrate	5.62 (dd)	50.0	44.2	201.8	0.687	0.00000
18	Uridine	4.36 (dd); 5.92 (d); 5.92 (d); 7.88(d)	66.1	65.2	125.7	0.562	0.00154
19	Adenosine	6.08 (d); 8.27 (s); 8.36 (s)	53.3	58.4	94.5	0.482	0.00394
20	6-hydroxynicotinate	6.62 (dd); 8.09 (d); 8.11 (m)	33.2	28.9	38.9	0.569	0.00118
21	Quinolinate	7.86 (dd); 8.02 (dd); 8.61 (dd)	38.2	47.2	148.2	0.638	0.00000
22	β-glucose	3.23-3.90 (m); 4.66 (d)	52.0	68.3	-90.5	0.805	0.00000
23	α-glucose	3.39-3.84 (m); 5.25 (d)	49.5	84.4	-90.9	0.820	0.00000
24	Trehalose	3.46 (m); 3.66 (m); 3.87 (m); 5.19 (d)	53.6	75.9	-60.8	0.652	0.00011
25	Choline	3.21 (s); 4.07 (m)	27.6	23.9	63.0	0.772	0.00000
26	GPC#	3.23 (s); 4.31 (m)	31.4	12.9	66.5	0.895	0.00000
27	Glycerol	3.46 (m); 3.65 (m); 3.87 (m)	29.8	24.8	116.5	0.823	0.00000
28	Lipids	0.80-0.92 (b); 1.25-1.41 (b)	31.8	44.7	338.1	0.793	0.00000
29	Gluconate	3.65(m); 3.81(m); 4.03(t); 4.13(d)	57.0	55.8	-11.8	0.187	0.53763

The differences in the percentages show higher or lower levels of metabolites in caps compared with stems. The *P* values were obtained using Mann-Whitney-Wilcoxon tests. The OPLS-DA correlation coefficients were obtained from a model with the following parameters: *R* ^2^ *X* = 0.774, *R* ^2^ *Y* = 0.938 and *Q* ^2^ *Y* = 0.904.

# NAC, *N*-acetylated compounds; GPC, glycerophosphocholine.

* s, singlet; d, doublet; t, triplet; m, multiplet; q, quadruplet; dd, doublet of doublets; bs, broad signal.

### The influence of topsoil on the metabolic composition of *A. muscaria* basidiocarp

**Figure 5:**
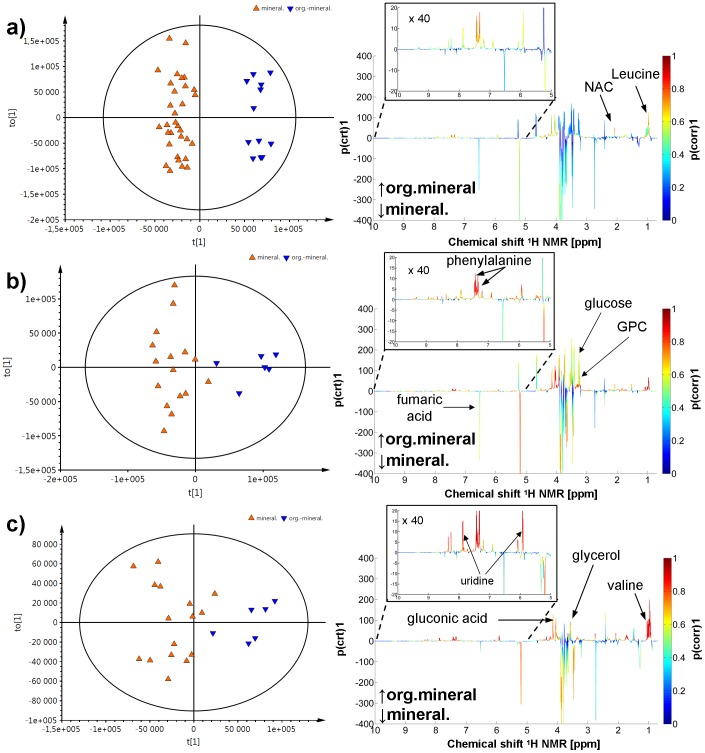
OPLS-DA score plots discriminating between samples from mineral and organic-mineral topsoil for: a) whole fruiting bodies (*R* ^2^ *X* = 0.924, *R* ^2^ *Y* = 0.944 and *Q* ^2^ *Y* = 0.790); b) stems (*R* ^2^ *X* = 0.814, *R* ^2^ *Y*  = 0.830, *Q* ^2^ *Y* = 0.728); and c) caps (*R* ^2^ *X* = 0.608 *R* ^2^ *Y* = 0.732, *Q* ^2^ *Y* = 0.554). The corresponding loadings from the OPLS-DA models are shown on the right. The color bar corresponds to the absolute value of the correlation loading.

The samples used in this study were collected from two types of topsoil, mineral and organic-mineral. Sixteen mushroom samples were taken from mineral topsoil, while six samples were collected from organic-mineral topsoil. Because *A. muscaria* stems and caps exhibit different metabolic compositions, the influence of the topsoil was studied separately in whole fruiting bodies, stems and caps. All three OPLS-DA models showed separation between samples originating from different topsoil types (mineral vs. organic-mineral) and were characterized with the following parameters: whole fruiting bodies (*R* ^2^ *X* = 0.924, *R* ^2^ *Y* = 0.944 and *Q* ^2^ *Y* = 0.790) (**Figure 5a**), stems (*R* ^2^ *X* = 0.814, *R* ^2^ *Y* = 0.830, *Q* ^2^ *Y* = 0.728) (**Figure 5b**), and caps (*R* ^2^ *X* = 0.608 *R* ^2^ *Y* = 0.732, *Q* ^2^ *Y* = 0.554) (**Figure 5c**). A general analysis of the corresponding loading plots (**Figure 5**) showed that the topsoil type dependence is of a different origin in stems and caps. Therefore, the identified metabolites were tested using univariate statistics, and the results for all three comparisons are reported in **Table 3**.

**Table 3:** Influence of mineral and mineral-organic topsoil on the metabolic composition of *A. muscaria* basidocarps.

No	Compound	Mineral vs. Organic-mineral								
		ALL			Stem			Cap		
		difference [%]	OPLS-DA absolute correlation coefficient	*P* value	difference [%]	OPLS-DA absolute correlation coefficient	*P* value	Difference [%]	OPLS-DA absolute correlation coefficient	*P* value
1	Isoleucine	−43.5	0.436	0.00298	−36.1	0.630	0.00032	−46.6	0.882	0.00335
2	Leucine	−58.5	0.629	0.00000	−58.4	0.952	0.00005	−58.6	0.915	0.00335
3	Valine	−52.6	0.400	0.04270	−28.3	0.489	0.00799	−57.0	0.873	0.00118
4	Alanine	−0.9	0.033	0.80496	24.4	0.090	0.29384	−6.1	0.418	0.69312
5	Aspartate	−15.0	0.211	0.10572	−21.6	0.600	0.03281	−9.9	0.429	0.29384
6	Asparagine	−28.9	0.358	0.00026	−38.3	0.806	0.00080	−21.1	0.670	0.01335
7	Threonine	−22.5	0.365	0.01231	−34.4	0.817	0.00032	−13.4	0.293	0.17750
8	Tyrosine	−50.6	0.637	0.00004	−55.6	0.738	0.00051	−47.0	0.575	0.01699
9	Phenylalanine	−51.6	0.743	0.00000	−48.3	0.935	0.00005	−54.4	0.923	0.00051
10	4-aminobutyrate	−22.0	0.099	0.03735	−27.0	0.433	0.03281	−18.7	0.306	0.15445
11	NAC#	−34.0	0.788	0.00002	−38.4	0.749	0.00335	−30.2	0.894	0.00051
12	Acetate	−12.6	0.139	0.20418	−8.5	0.253	0.44941	−15.6	0.238	0.29384
13	Succinate	−5.9	0.012	0.57583	6.5	0.025	0.85773	−16.5	0.263	0.17750
14	Malate	55.1	0.575	0.00048	70.0	0.529	0.00335	39.2	0.409	0.04018
15	Fumarate	78.8	0.255	0.07070	104.6	0.418	0.04873	29.7	0.101	0.29384
16	Formate	20.1	0.186	0.45780	14.8	0.191	0.23079	29.5	0.319	0.69312
17	UDP-carbohydrate	32.0	0.195	0.47381	−31.4	0.330	0.15445	73.8	0.383	0.04873
18	Uridine	−59.8	0.583	0.00009	−58.3	0.755	0.00080	−60.5	0.896	0.00051
19	Adenosine	−53.1	0.587	0.00043	−50.7	0.701	0.00335	−54.3	0.892	0.00608
20	6-hydroxynicotinate	12.5	0.048	0.35437	−16.8	0.430	0.13352	44.2	0.020	0.00608
21	Quinolinate	36.6	0.196	0.52358	−10.1	0.213	0.64067	66.6	0.231	0.02662
22	β-glucose	−39.3	0.241	0.16062	−41.3	0.568	0.02134	−13.2	0.166	0.09803
23	α-glucose	−33.6	0.221	0.82515	−38.6	0.558	0.02134	95.3	0.302	0.11481
24	Trehalose	250.9	0.503	0.00003	204.6	0.794	0.00118	455.5	0.727	0.00019
25	Choline	10.8	0.108	0.35437	−9.0	0.211	0.97136	26.3	0.248	0.07010
26	GPC#	−10.4	0.124	0.36820	−35.0	0.735	0.00080	11.0	0.099	0.13352
27	Glycerol	−21.3	0.272	0.17714	−20.7	0.554	0.23079	−21.5	0.784	0.04018
28	Lipids	2.7	0.023	0.42669	−28.1	0.568	0.07010	12.3	0.263	0.69312
29	Gluconate	−46.1	0.561	0.00026	−48.8	0.705	0.00080	−41.7	0.750	0.03281

The differences in percentages show the higher or lower levels of metabolites in *A. muscaria* basidocarps growing on mineral topsoil compared with those growing on mineral-organic topsoil. The *P* values were obtained using Mann-Whitney-Wilcoxon tests. OPLS-DA correlation coefficients were obtained for the following tissues: whole fruiting bodies (*R* ^2^ *X* = 0.924, *R* ^2^ *Y* = 0.944 and *Q* ^2^ *Y* = 0.790), stems (*R* ^2^ *X* = 0.814, *R* ^2^ *Y* = 0.830, *Q* ^2^ *Y* = 0.728), and caps (*R* ^2^ *X* = 0.608 *R* ^2^ *Y* = 0.732, *Q* ^2^ *Y* = 0.554).

# NAC, *N*-acetylated compounds; GPC, glycerophosphocholine.

Nine metabolites were found in similar concentrations in the two types of topsoil namely: alanine, acetate, succinate, formate, UDP-carbohydrate, 6-hydroxynicotinate, quinolinate, choline and lipids. *A. muscaria* basidocarps from mineral topsoil exhibited lower concentration of isoleucine, leucine, valine, asparagine, tyrosine, phenylalanine, NACs, uridine, adenosine and gluconate in comparison to basidocarps from organic-mineral topsoil. Only two metabolites, malate and trehalose, were present at higher concentrations in mineral topsoil compared to organic-mineral topsoil. There was only one compound - glycerol - among the identified metabolites that changed significantly in caps (*P* value <0.05 and OPLS-DA correlation coefficient *p(corr)*>0.4) but not in stems. In contrast, stems exhibited a strong dependence on the soil type, showing significantly different levels of seven metabolites that correlated with the topsoil type, whereas there were no differences in the levels of these metabolites in caps that correlated with the topsoil type. The metabolites at significantly lower levels in *A. muscaria* stems in mineral topsoil compared to organic-mineral topsoil were aspartate, threonine, 4-aminobutyrate, α- and β-glucose and GPC, while only the level of fumarate was elevated.

### Correlation analysis of topsoil composition and *A. muscaria* metabolites

**Figure 6:**
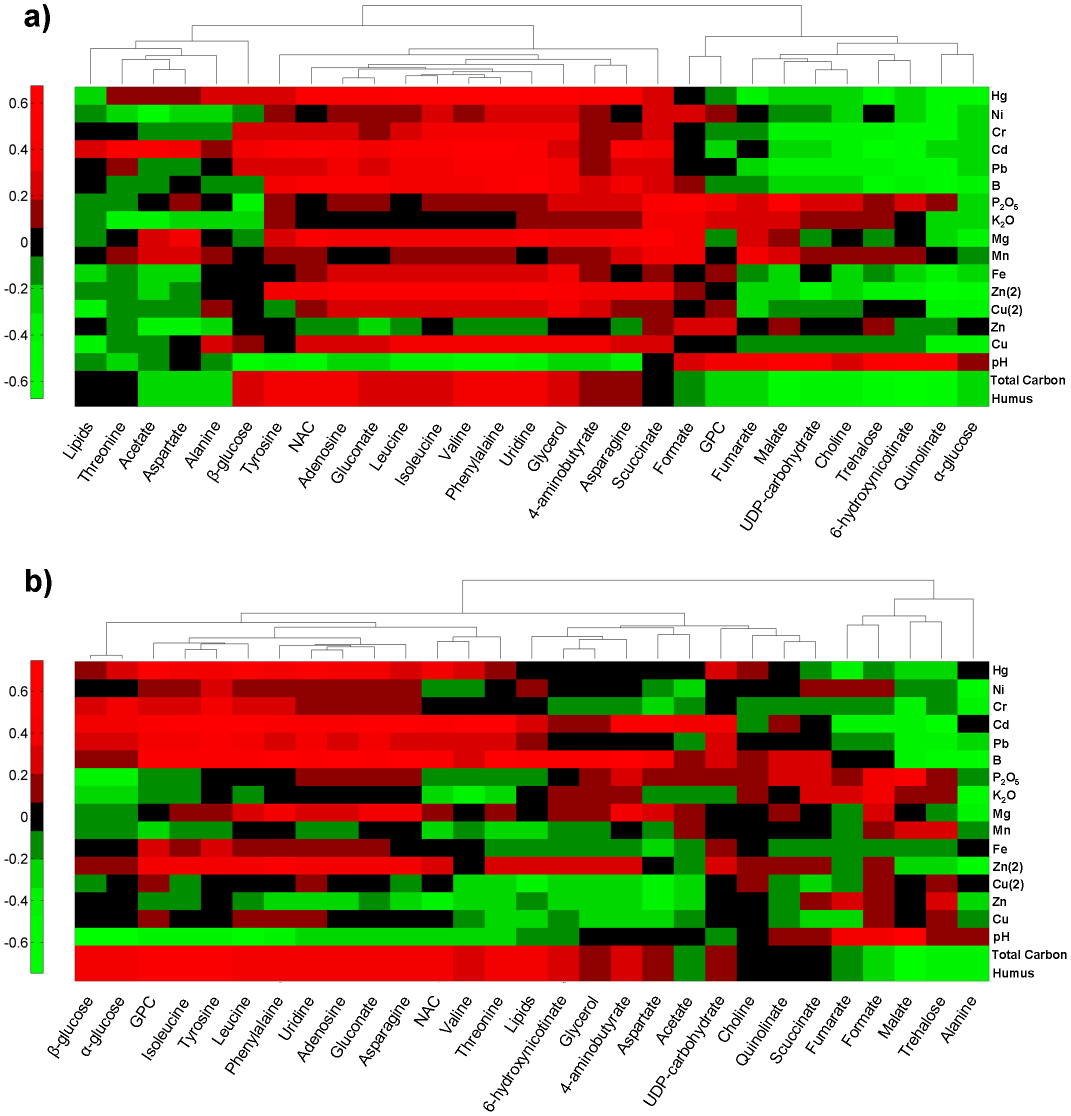
Heat map showing the spearman rank coefficients calculated between soil properties and metabolites in *A. muscaria* basidocarp: a) in caps; b) in stems. The B, P_2_O_5_, Mg, Zn(2) and Cu(2) were determined as available elements, while Hg, Ni, Cr, Cd, Pb, Mn, Fe, Cu and Zn were assayed as the general concentration in dry matter.

To discover which topsoil properties influence the metabolic composition of *A. muscaria* fruiting bodies, a correlation analysis was performed. To better evaluate the biological meaning of the results, the selected topsoil properties were analyzed using the following order: toxic heavy metals, metalloids, non-metals, essential metals, pH and type of soil. The results were visualized using a heat map approach (**Figure 6**). Clustering was applied only for columns (metabolites) since rows (topsoil properties) remained in established order.

In caps clustering revealed evident correlation patterns. Metabolites such as: β-glucose, tyrosine, NAC, adenosine, gluconate, leucine, isoleucine, valine, phenylalanine, uridine, glycerol, 4-aminobutyrate, asparagine and succinate exhibited positive correlations with majority of soil parameters (with exception of pH and total Zn). Contrary, malate, UDP-carbohydrate, choline, trehalose, 6-hydroxynicotinate, quinolinate and α-glucose showed negative correlations regarding to topsoil parameters with exception of P_2_O_5_, K_2_O, Mn contents and soil pH.

On the contrary in stem the correlation patterns were less evident. For example, different correlation patterns were observed for caps and stems with respect to α- and β-glucose. In stems both glucose forms were clustered together, while in caps presented different behavior. In stems β- and α-glucose, GPC, isoleucine, tyrosine, leucine, phenylalanine, uridine, adenosine, gluconate and asparagine had exhibited mostly positive correlations with heavy metals and boron. On the other hand, NAC, valine, threonine and lipids showed positive correlations with Cd, Pb and B. Moreover total carbon revealed the same pattern as humus in both parts of mushrooms. Those topsoil properties demonstrated more positive correlations in case of stems than caps.

## Discussion

The different distributions of metabolites in *A. muscaria* basidocarp caps and stems reflect the diverse functions of these parts. In general, stems serve two purposes. First, they support the cap in spore dispersal, and second, they allow the flow of water and solutes from the mycelium growing underneath the ground to the developing fruiting body [47], [48]. Our results shows that stems can fulfill storage functions, being reservoirs of energetic metabolites (trehalose, α- and β-glucose) and the accompanying TCA cycle-related compounds malate and fumarate. This result confirms the common knowledge that disaccharides and polysaccharides are especially good carbon sources for the production of fruiting bodies [47], [49]. Formate, which is present in stems, can be used in many biochemical pathways, e.g., puryvate, glyoxylate and dicarboxylate metabolism.

Most of a mushroom's energy is directed into cap development. Therefore, the elevated levels in almost all detected amino acids (except tyrosine and phenylalanine) in caps are likely due to an increased protein biosynthesis (anabolism and catabolism). Lipids are necessary to form cell membranes. Higher amounts of choline, GPC and glycerol fulfill the demand for lipid biosynthesis. Cholines are efficient methyl group carriers that can be utilized for lipid chain elongation. Furthermore, glycerol is a backbone of various lipids; thus, GPC most likely serves as a source of both glycerol and phosphate for phospholipid biosynthesis. Finally, acetate is a basic compound that transfers methyl groups by the formation of acetyl-CoA; therefore, its increased level in caps suggests intensified lipid biosynthesis. In addition, the higher concentrations of uridine and adenosine may reflect the higher reproductive rates of cap cells compared to stems in mature *A. muscaria* basidocarps. All of these compounds are necessary to maintain the functions of the cap as well as the entire fruiting body [50]. The presence of quinolinate molecules is especially interesting. These compounds might be responsible for the properties of the defined hallucinogenic substances, i.e., ibotenic acids and muscimol, that are responsible for the neurotoxic effect of *A. muscaria* [51].

The stronger dispersion observed for stem samples in the PCA score plot (**Figure 2a**) may have occurred for several reasons. One possibility is that there are different PBS extraction efficiencies in stems and caps. However, it is more likely that stems are more sensitive to external (physiological) stimuli than caps. This result may be attributable to the environmental factors, i.e., the type of soil and metal ions, that influence stems.

Indeed, seven metabolites were found at significantly different levels between the two topsoil types only in stems. The concentrations of α- and β-glucose in *A. muscaria* stems depended on the topsoil type, which is likely related to total carbon and humus content. Furthermore, those changes were related to the levels of aspartate, threonine, 4-aminobutyrate, fumarate and GPC.

Fungi cannot obtain energy directly from the sun but rather use the energy stored in decomposing plant and animal biomass to create their own mass. Interestingly, in topsoil with lower total carbon and humus contents, both stems and caps exhibited much higher trehalose concentrations (200% and 455%, respectively) than in *A. muscaria* from organic-mineral topsoil. This result may indicate that when basic nutrients are not available, mushrooms switch metabolism strategies and store energy in the form of abiotic stress modulator - trehalose.

Because fungi require certain metallic and non-metallic elements to meet their nutritional requirements [52], [53], metal ions in the soil may influence their biochemical pathways [53], [54]. In the present study, we observed such an indirect influence. Therefore the strongest effect on the metabolic composition of *A. muscaria* basidocarp must come from the topsoil humus and total carbon content, we observed many strong correlations between mushroom metabolites and inorganic topsoil elements. The concentrations of diverse metal ions in various mushrooms are described in the literature [55]-[62]. Mushrooms may bioaccumulate some toxic elements, which are considered as the main source of environmental pollution [63].

Our study cannot determine which biochemical pathways are influenced by the metal and non-metal ions present in topsoil. However, it shows that this effect most likely exists. Thus, it is important to examine metabolic studies in a wider context, including studies on nutrient sources, especially when studying mushrooms. Our results clearly show that in discovering new mushroom natural products or studying their metabolic pathways, topsoil properties should always be considered.

## Conclusions

This metabolomic study on *Amanita muscaria* revealed different metabolite contents depending on the morphological part of the fruiting body. Moreover, it is possible to distinguish mushrooms depending on the topsoil type (mineral vs. organic-mineral). Stems were found to be more sensitive to topsoil composition than caps. Correlations between topsoil elements and particular metabolites were found, showing the usefulness of holistic metabolomic approaches in the delineation and characterization of mushroom microenvironments and suggesting that topsoil mineral compositions influence the metabolic pathways of *A. muscaria*. In conclusion, in the discovery of new mushroom natural products or the study of mushroom metabolic pathways, topsoil properties should always be considered. Further studies in different regions should be performed using this species to determine the metal ion content in the soil as well as in mushroom fruiting bodies and to examine these data and with a metabolomics-based comprehensive approach.

## Supporting Information

Table S1 **Samples characteristics.**
(DOC)
