# Supplementary material for: Do Differences in Chemical Composition of Stem and Cap of Amanita muscaria Fruiting Bodies Correlate with Topsoil Type?
Source: PLoS One. 2014 Dec 1;9(12):e104084. doi: 10.1371/journal.pone.0104084 (PMC4249817; doi:10.1371/journal.pone.0104084)
Supplement: Table S1 — Samples characteristics. (DOC) [file pone.0104084.s001.doc]

Table S1. Samples characteristics.

| **Collection** | **Sample mass [mg]** | |
| --- | --- | --- |
| **number** | **Stem** | **Cap** |
| **96** | 81,27 | 83,54 |
| **98** | 84,21 | 83,78 |
| **102** | 83,49 | 81,96 |
| **105** | 83,91 | 83,66 |
| **129** | 84,70 | 82,20 |
| **131** | 83,55 | 83,82 |
| **120** | 83,87 | 81,41 |
| **121** | 80,31 | 82,13 |
| **275** | 85,18 | 82,90 |
| **278** | 83,79 | 84,31 |
| **280** | 85,00 | 81,60 |
| **136** | 83,37 | 80,40 |
| **138** | 81,90 | 82,10 |
| **141** | 83,79 | 84,50 |
| **82** | 82,78 | 82,70 |
| **83** | 80,48 | 84,61 |
| **72** | 83,45 | 81,27 |
| **73** | 79,87 | 82,09 |
| **75** | 81,36 | 84,33 |
| **161** | 86,50 | 80,90 |
| **165** | 83,46 | 83,82 |
| **169** | 84,24 | 79,50 |
